# BCG therapy is associated with long-term, durable induction of Treg signature genes by epigenetic modulation

**DOI:** 10.1038/s41598-021-94529-2

**Published:** 2021-07-22

**Authors:** Ryan C. Keefe, Hiroyuki Takahashi, Lisa Tran, Kacie Nelson, Nathan Ng, Willem M. Kühtreiber, Denise L. Faustman

**Affiliations:** 1 Immunobiology Laboratories, Massachusetts General Hospital, Bldg 149, 13th Street, Boston, MA 02116 USA; 2 Harvard Medical School, Boston, MA 02116 USA; 3 Alles Research Institute, Massachusetts General Hospital, Boston, MA 02116 USA

**Keywords:** Microbiology, Vaccines, Live attenuated vaccines

## Abstract

Induction of immunosuppressive T-regulatory cells (Tregs) is a desirable goal in autoimmunity, and perhaps other immune diseases of activation. One promising avenue is with the bacille-calmette-guérin (BCG) vaccine in autoimmune type 1 diabetes (T1D). Its administration is associated with gradual clinical improvements in human autoimmunity over a 2–3 year post-vaccination period. We hypothesize that those improvements, and their unusually long time course to fully materialize, are partially attributable to BCG’s induction of Tregs. Here we report on a 3 year-long longitudinal cohort of T1Ds and examine the mechanism by which Treg induction occurs. Using the Human Infinium Methylation EPIC Bead Chip, we show that BCG vaccination is associated with gradual demethylation of most of 11 signature genes expressed in highly potent Tregs: Foxp3, TNFRSF18, CD25, IKZF2, IKZF4, CTLA4, TNFR2, CD62L, Fas, CD45 and IL2; nine of these 11 genes, by year 3, became demethylated at the majority of CpG sites. The Foxp3 gene was studied in depth. At baseline Foxp3 was over-methylated compared to non-diabetic controls; 3 years after introduction of BCG, 17 of the Foxp3 gene’s 22 CpG sites became significantly demethylated including the critical TSDR region. Corresponding mRNA, Treg expansion and clinical improvement supported the significance of the epigenetic DNA changes. Taken together, the findings suggest that BCG has systemic impact on the T cells of the adaptive immune system, and restores immune balance through Treg induction.

## Introduction

Exposure to chronic infectious diseases can result in increases in host Treg cells^1,2^. The Treg and infectious disease link is thought to result from the co-evolutionary existence of pathogens developing mechanisms for long-term survival within host cells without host-immune recognition. In tuberculosis, the granuloma is surrounded by host-induced Treg cells. Infection with tuberculosis (*Mycobacterium* tuberculosis), as well as leprosy (*Mycobacterium* leprae), is accompanied by an increase in circulating Treg cells^3,4^ Mice experimentally infected with tuberculosis show expansion of Tregs^5^.

Induction of Treg cells, in numbers or activation state, is a one type of therapeutic approach in human inflammatory diseases. One possible way to increase the numbers or function of Tregs is to use microorganisms that typically with infection already uses this mechanism for host immune escape. Strategic introduction of long-lived organisms that are not harmful to hosts, such as the attenuated *Mycobacterium* bovis of the BCG vaccine, might be beneficial for host Treg induction. Increasing Tregs numbers or function maybe one solution to inflammation. Underlying defects in Treg function, sometimes related to Foxp3 expression or function, are common in severe human immune diseases. The first disorder associated with dysfunction of the Foxp3 gene impacting Tregs, is IPEX syndrome (immunodysregulation polyendocrinopathy enteropathy X-linked). This syndrome of autoimmune driven diarrhea, eczema and often type 1 diabetes, is caused by a multitude of now identified germline mutation in the Foxp3 gene with over 70 mutations^6^. Subsequent studies support the Foxp3 gene as the defining protein of the Treg cellular lineage and identify potent Tregs with suppressive function^7^. The Foxp3 gene works in concert with other signature genes of potent Tregs. In other immune diseases, specific genetic mutations in single Treg-associated genes have not been identified, suggesting other genetic mechanisms for the poor Treg function in inflammatory diseases. This paves the way for alternative therapeutic approaches with quantitative defects to overcome the lackluster performance of Tregs in autoimmunity.

The BCG vaccine is being increasingly used in autoimmune clinical trials as an anti-inflammatory drug^8–13^. BCG vaccines are also being used for combatting childhood asthma and allergies^14–16^. Evidence exists especially in at least two human autoimmune diseases, multiple sclerosis (MS) and type 1 diabetes (T1D), of BCG’s therapeutic effects manifesting gradually over a post-infection period of 3 or so years^10,12^. In autoimmune diabetic subjects, 8 weeks after two BCG vaccinations, CpG sites in Treg signature genes have shown statistically significant but small early demethylation changes^12^. This, along with corresponding increases in mRNA, supports increased Treg function to control inflammation. Although this time course is short, similarly short time courses with BCG therapy also suggest, at the protein level, that inflammatory cytokines expressed for 2 or 3 months are also changing which could be a consequence of BCG impact on cytokines produced by monocytes^16^. Long-term and multi-year effects of BCG vaccines for durable boosting of the Treg response have not been studied. A vaccine with an avirulent version of the microorganism might show benefit by Treg induction if these benefits could be sustained on a multi-year basis. BCG’s apparent control over Treg signature genes, including Foxp3, appears to be a path to understanding its clinical success in multiple human inflammatory diseases.

Traditionally the BCG vaccine has been thought to have influence over the innate immune response, meaning lasting changes predominantly in host monocytes. The innate immune response of BCG on the host is certainly controlled in part by the histone activity of key genes involved in cytokine secretion^18–20^. The methylation changes in monocytes have been coined “trained innate immunity”. BCG’s impact on the host immune response may also involve the adaptive immune system. Indeed the impact of BCG on glycolysis can be monitored both within T cells and monocytes suggesting adaptive immune changes from BCG vaccine therapy^12,13,17^. The MYC pathway for glycolysis after BCG vaccinations is equally induced in T cells as well as monocytes by methylation and downstream reciprocal mRNA changes^20^. Data in mice supports the concept that long-lasting BCG immune effects can be driven by infection of host bone marrow stem cells resulting in presumed multi-lymphoid lineage effects^21^. These effects support the concept of BCG’s therapeutic reach extending beyond innate immunity. Since T cells are part of the adaptive immune response, a long-term examination of the CD4 Treg function after BCG vaccination could support the broader utilization of short-term and long-term immune alterations.

It is appreciated that epigenetic mechanisms regulate T cell responses during T cell development^22–24^. Traditionally Treg numbers have been studied at the level of surface markers with flow cytometry methods, but these methods are cumbersome and are best studied in freshly harvested cells. Although CD4 + T cells expressing intracellular Foxp3 are almost always studied, other studies have focused on the surface expression of CD45 (naïve RA and memory RO) cells and CD25 surface protein on Tregs as additional markers of potency. Potent Treg cells also express high densities of TNFR2. In T1D there are defects in both the abunbance of potent Treg cells with TNFR2 and CD45RO expression^25–29^. It has been proposed that poor numbers and function of Tregs in T1D can be corrected with antibody agonism of the TNFR2 receptor^29^. DNA methylation patterns in T1D may mediate genetic risk to disease^26^.

DNA methylation patterns may mediate the plasticity of human Tregs during development, but also BCG vaccine therapy may also have impact on the Treg signature genes, also through methylation changes. Unlike cytokine therapy, a durable and long lasting BCG effect on the Tregs through the Treg Foxp3 gene or the CD25 receptor (IL2 receptor) has the potential to permanently change methylation thereby resulting in the desired stability and durability of this microorganism and host approach. This longitudinal human cohort study of 3 years duration, BCG therapy is associated with induction of most of 11 signature genes of potent Tregs: Foxp3, TNFRSF18, CD25, IKZF2, IKZF4, CTLA4, TNFR2, CD62L, Fas, CD45 and IL2. A central question underlying the use of BCG as a method to reset the immune system by Treg induction is whether its benefits are durable and can it work in a disease setting of autoimmunity. The safety of the BCG vaccine has long been confirmed for over 125 years of continuous use around the globe. This is the first long-term study of methylation patterns of Treg signature genes that might be influenced by host microbe interactions of the BCG organism.

## Results

### Human studies of repeat BCG vaccinations in subjects with type 1 diabetes investigate the induction of Treg cells

In this cohort study we ask the following question: can BCG vaccination induce long-term epigenetic changes in Treg signature genes? Studying the epigenetics of Treg biology over a 3-year time period was compared to mRNA changes to see if BCG changed methylation in a meaningful way to impact gene expression. Throughout these studies, in most cases the Human Infinium Methylation assay is used to study these Treg signature genes: FOXP3, TNFRSF18, CD25, IKZF2, IKZF4, CTLA4, TNFR2, CD62L, FAS, CD45 and IL2. For the Treg specific demethylation region (TSDR) of the Foxp3 gene an alternative method was used since the Infinium assay did not contain this DNA region.

### BCG vaccine therapy over 3 years gradually demethylates most Treg signature genes

**Figure 1:**
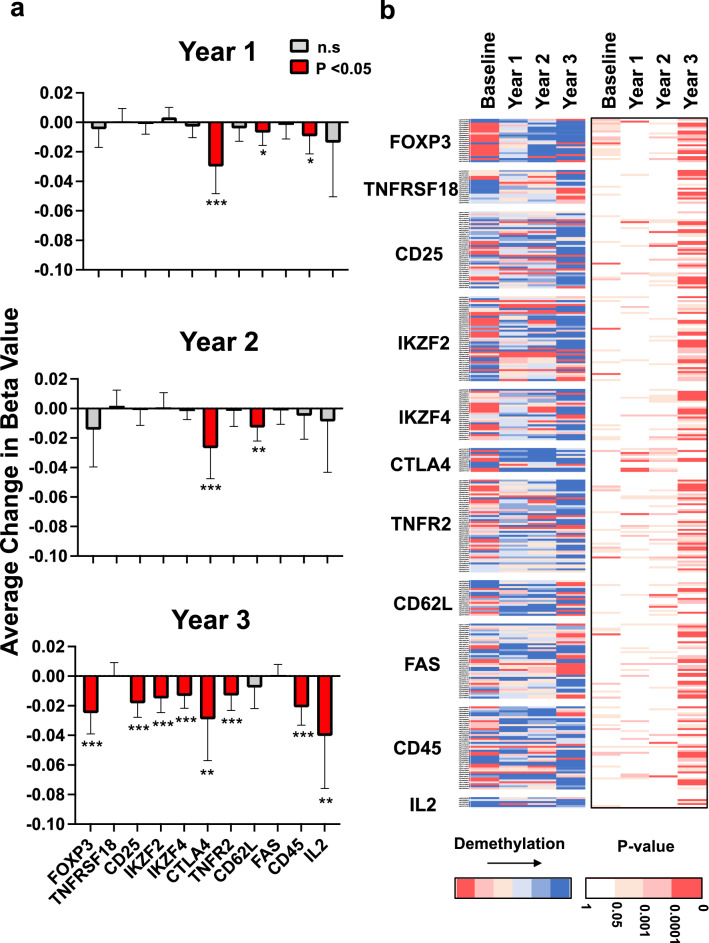
Most Treg signature genes gradually demethylate over a three year time course after in vivo BCG vaccinations. Methylation changes in eleven well-known Treg signature genes were quantified yearly after multi-dose BCG vaccine therapy (n = 13 type 1 diabetic subjects). (**a**) The methylation values are current methylation values at year 1, year 2 or year 3 compared to baseline expressed as average change in beta values. Positive beta values indicate increased methylation and thus decreased gene expression; negative methylation values indicate demethylation and increased gene expression. Significant decreases in methylation after BCG were seen in eight of 11 genes: FOXP3, CD25, IKZF2, IKZF4, CTLA4, TNFR2, CD62L, CD45 and IL2 (p value < 0.05: *; p value < 0.01: **; p value < 0.001: ***). Red bars represent statistically significant change in methylation. (**b**) The left heatmap shows average difference in beta values for individual Illumina CG Identifiers (CGs) of the Treg signature genes at yearly intervals. Increased blue coloration in the heat map means increased demethylation of the CpG sites within a Treg signature gene. The right heatmap indicates the p-values in the corresponding CpG sites; Blank and red indicate without or with significance, respectively, and degree of red color reflects the strength of significance. In the both heatmaps each individual CpG gene site is represented as a single bar.

We analyzed the methylation state of the CG Probe IDs (CpGs) for the eleven signature genes of Treg cells (Fig. 1a). The data are presented as the overall demethylation per gene (Fig. 1a) and the heat map data of all individual CpG sites of each gene over the same 3-year time period compared to baseline (pre-vaccination) (Fig. 1b). We present calculated average beta values across all CpGs for each gene separately at yearly timepoints and calculate changes compared to baseline (Supplementary Tables). Individual CpG sites per gene are also presented as a heatmap of color coding, with blue representing greatest demethylation.

As compared to baseline, most of the signature genes demethylate after BCG therapy. For these genes—FoxP3, IKZF4, CTLA4, TNFR2, CD62L and CD45, IL2—the demethylation patterns are already significant at year 1 (red bars) but then become even more pronounced by year 3. For the CD25 and IKZF2 genes, demethylation also occurs but was not observed until year 3. TNFRSF18, FAS do not show significant differences. These methylation studies support BCG as a possible in vivo method to decrease methylation of Treg signature genes.

Similar to the overall demethylation patterns of entire genes, it is also apparent that individual CpGs for the 11 signature genes are also demethylating (more blue) over the yearly intervals and demethylation continued at least up to year 3 (Fig. 1b). These data suggest BCG vaccine-induced modulations of the Treg cell are at the DNA level (by changing demethylation) and are a slow and gradual process that mirrors the time course of clinical improvement, i.e. lowering of the HbA1c for diabetes and halt of CNS pathology in multiple sclerosis^10,12^.

Simultaneously, we accessed the overall methylation pattern of CpGs in CD4 + T cells. In total 697315 CpG sites were evaluate. Approximately half of CpG sites were hyper- or hypomethylated in T1D at baseline compared to NDC (Supplementary Fig. S2). On the other hand, after BCG vaccination, the epigenetics tended to be hypomethylated at year 1 and 2, and the trend became pronounced at year 3 (Supplementary Fig. S2). Thus, BCG microbe seems to demethylate the many of the immune related CpG sites in T1D CD4 + T cells and this patterning was not associated with over-methylation DNA trends with 3 years of monitoring.

### T1Ds have excessive methylation of the Foxp3 gene; this over methylation defect is corrected by multi-dose BCG therapy at most of the gene’s CpG sites

**Figure 2:**
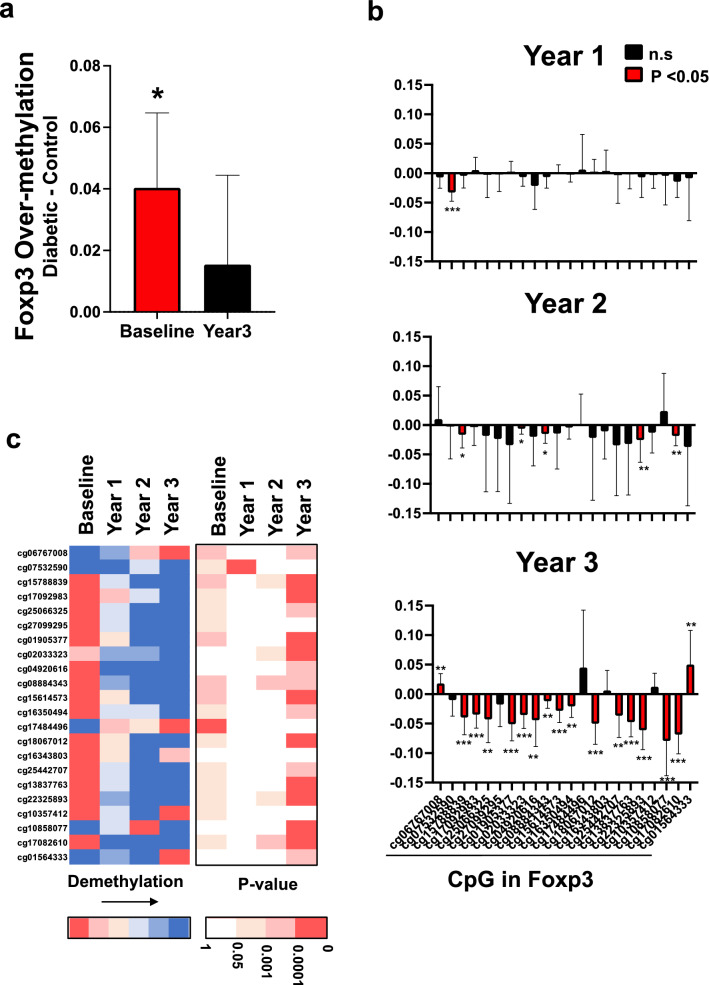
Baseline over methylation of the Foxp3 gene of Treg cells compared to controls; BCG therapy gradually restores towards normal methylation patterns over three years. (**a**) At baseline, prior to BCG treatment, type 1 diabetic subjects (n = 13) have over methylation of the Foxp3 Treg gene compared to non-diabetic control subjects (n = 8) suggesting inadequate Treg mediated suppression, a trait of autoimmunity (p = 0.03). At three year follow-up after multi-dosing with BCG therapy, the type 1 diabetic subjects’ over methylation defect in the Foxp3 gene corrects towards normal and is no longer statistically different from the degree of methylation in non-diabetic control cells (p = 0.39). Data is expressed as beta values of the difference between diabetic and control methylation. The data for each CpG is color coded separately (p value < 0.05: *; p value < 0.01: **; p value < 0.001: ***). Red bars represent statistically significant change in methylation. (**b**) Methylation changes for the individual CpG sites of Foxp3 gene after BCG were quantified compared to self at baseline. Average yearly changes in Beta Values of Foxp3 CpG sites in CD4 + cells of patients given BCG (n = 13). Beta value differences were calculated for each patient relative to baseline. (**c**) The left heatmap shows average difference in beta values for individual Foxp3 CpGs sites at yearly intervals. Increased blue coloration in the heatmap means increased demethylation of the CpG sites within the Foxp3 gene. The right heatmap indicates the p-values in the corresponding CpG sites; Blank and red indicate without or with significance, respectively, and degree of red color reflects the strength of significance.

Foxp3 is arguably the most important marker gene for Treg cells. It is well-known from flow cytometry studies that Treg cells have a CD4 +, FOXP3^high^, CD25^high^ phenotype, and these cells might show improvements in overall cell numbers or potency early after BCG vaccinations^12^. Here we change the method of analysis to see if BCG might be mechanistically working at the level of host DNA and changing the methylation patterns of CpG sites of the Foxp3 gene that controls Treg potency and function. At baseline (before BCG introduction) we show that Foxp3 gene methylation is over-methylated in T1D DNA samples (n = 13) compared to non-diabetic control DNA samples (n = 8); this is consistent with Tregs not having fully potent function (Fig. 2a). We also show that at year 3, the diabetic has less overall Foxp3 methylation than earlier, a pattern indicating a correction towards normal because the statistically significant difference is eliminated (Fig. 2a).

We next examined the 22 CpG sites of the human Foxp3 gene and studied the patterns of change in type 1 diabetics compared to self at baseline prior to a BCG vaccination over the 3 year observation period (Fig. 2b). Using the Infinium Methylation EPIC Bead Chip Kit, which includes 22 CGs for Foxp3, we isolated genomic DNA (gDNA) from CD4 + T cells (n = 13 patients) and determined beta values for all Foxp3 CGs at baseline (before BCG treatment) and at yearly intervals after BCG therapy. The average difference in beta values for each CpG are shown in the heatmap. Each CpG was color-coded separately. The heatmap shows that demethylation of several CG starts in year 2, and the majority of CGs are demethylated by year 3 (17 out of 22 CGs are colored blue) (Fig. 2c).

In the graphs, the individual CpGs were averaged across the 13 T1D patients at each timepoint and then displayed as the average change in beta value as compared to baseline. The graphs show the progression of demethylation of the Foxp3 CGs over time. At year 1 only one CG was significantly different from baseline, whereas the number of significant CGs increased to 5 by year 2 and to 17 by year 3; a total of 22 Foxp3 CpG sites were studied. The great majority of CpGs show progressive demethylation. Demethylation of a gene usually results in increased transcription of the gene and thus usually results in increased protein.

### Type 1 diabetic subjects with early (< 20 years) versus later (> 20 years) age of onset have different trends in the rate of Foxp3 gene demethylation over the 3 year time course

**Figure 3:**
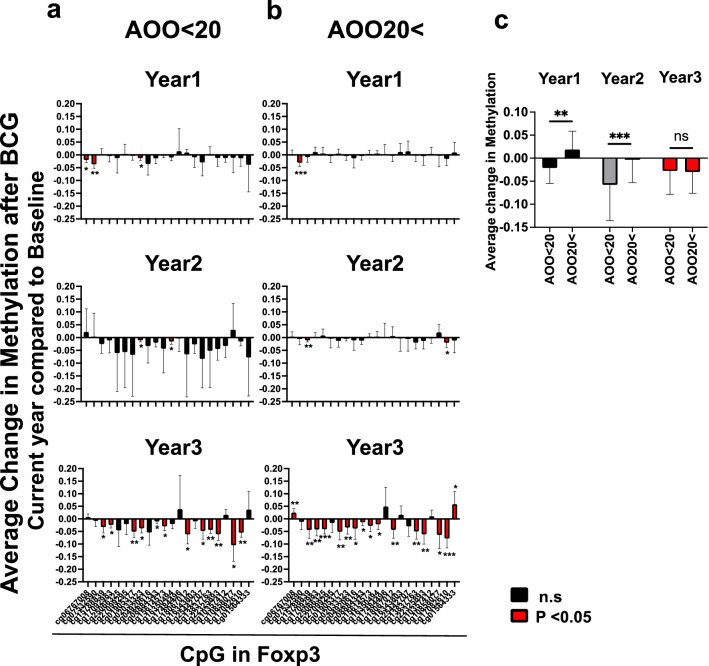
Younger Age of onset of diabetic subjects appear to show a different rates of demethylation at the CpG sites of Foxp3 gene. Type 1 diabetic subjects with a young age of onset (AOO) are considered to have a more serious and aggressive disease with rapid destruction of the pancreatic insulin secreting cells often defined as AOO < 20 years (**a**, left column, n = 5). Type 1 diabetic subjects with an older age of onset (AOO > 20) years are considered to have a more indolent disease with onset at older ages (**b**, right column, n = 8). This in the clinic correlates with a faster lowering of blood sugars after BCG therapy^10^. Both groups of type 1 diabetics with varying ages of onset had a BCG response as related to Foxp3 demethylation, but early AOO type diabetics (AOO < 20) had an apparent faster responding trend over the 3 year monitoring time period. **c**. Average change in beta value of Foxp3-related CpG sites are shown at AOO < 20 and 20 <. At year 1, the demethylation patterns were statistically different between early and late onset with early onset diabetic subjects showing faster demethylation (p = 0.009). At year 2 the demethylation patters were again statistically different with the early onset diabetic subjects showing faster Foxp3 gene demethylation (p = 0.0005) but by year 3 both clinical subjects with type 1 diabetics but varying is the disease onset had achieved equal demethylation of the Foxp3 gene (p = 0.64). Right column indicates the difference in beta value between AOO < 20 and 20 <. Red bars represent statistically significant changes in methylation vs baseline for individual CpGs.

Age of onset of diabetes is often considered an indication of the aggressiveness of the autoimmune disease. Indeed the pancreatic insulin cell rate of decline from the autoimmune attack is faster if the age of onset is early; if type 1 diabetes occurs at later times in life the destruction of the pancreas occurs at a much slower rate, often measured by the decline in C-peptide^30^. Here we define early onset diabetes as childhood diabetes with an age of onset < 20 years (n = 5, Fig. 3a); older onset is defined as disease onset > 20 years of age (n = 8, Fig. 3b). Although the sample sizes of these groups were small, trends were observed when the two groups were directly compared (Fig. 3c). At year 1, the demethylation patterns were statistically different between early and late onset with early onset diabetic subjects showing faster demethylation (p = 0.009). At year 2 the demethylation patters were again statistically different with the early onset diabetic subjects showing faster Foxp3 gene demethylation (p = 0.0005) but by year 3 both clinical subjects with type 1 diabetics but varying is the disease onset had achieved equal demethylation of the Foxp3 gene (p = 0.64).

### CD25, a central gene in Treg suppression shows 3 years of gradual CpG demethylation across the majority of its CpG sites.

**Figure 4:**
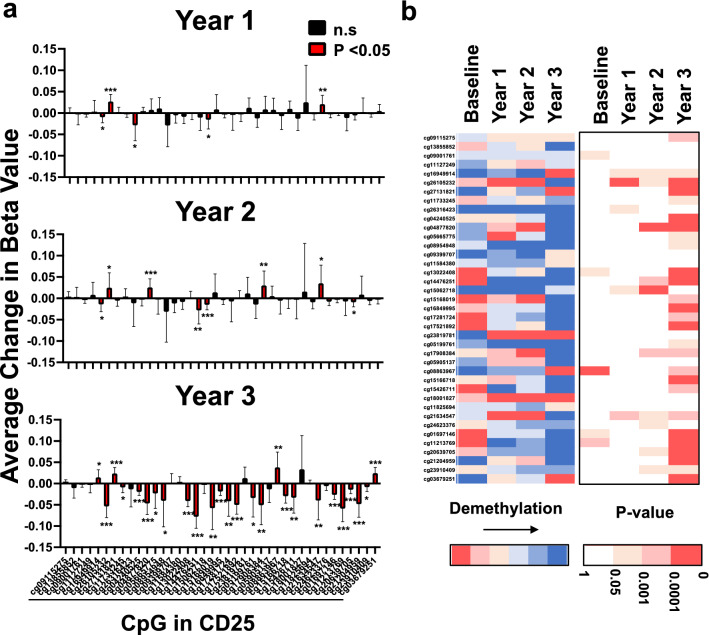
The Treg associated CD25 gene (IL2 receptor) of potent Treg cells after in vivo BCG treatment shows gradual, significant, and a three year decreases in methylation. (**a**) Methylation changes for the individual CpG sites (n = 39) of the CD25 gene after BCG vaccine therapy were quantified compared to baseline. Average yearly changes in Beta values of CD25 CpG sites is shown at Year 1, Year 2 and Year 3 for multi-dosed type 1 diabetic subjects (n = 13). Beta value differences were calculated for each patient relative to baseline. The data for each CpG is color coded separately (p value < 0.05: *; p value < 0.01: **; p value < 0.001: ***). Red bars represent statistically significant change in methylation. (**b**) The heat map shows average difference in Beta values for individual CD25 CpGs sites at yearly intervals. Increased blue coloration in the heatmap means increased demethylation of the CpG sites within the CD25 gene. The right heatmap indicates the p-values in the corresponding CpG sites; Blank and red indicate without or with significance, respectively, and degree of red color reflects the strength of significance.

In addition to Foxp3^high^ , Tregs also feature a CD25^high^ phenotype with high surface expression in potent Tregs. A high phenotype means by flow cytometric studies the density and thus the overall expression of the CD25 protein is high. CD25 is also known as IL2R (IL2 receptor). We therefore analyzed changes in methylation of the CpGs of the CD25 gene (Fig. 4). As shown for the Foxp3 gene, we also found progressive demethylation of CD25 CpGs over time, with the greatest demethylation differences vs baseline occurring by year 3 (29 out of 39 CpG demethylated at year 3, out of which 22 reached statistical significance. This is also reflected in the heatmap (Fig. 4b). The data shows that the greatest number of demethylated CpGs of CD25 are present at year 3, i.e., most CpG sites at year 3 have a dark blue color.

### CTLA4 gene after BCG treatment shows early demethylation and de-methylation of a CpG site associated with diabetes risk

**Figure 5:**
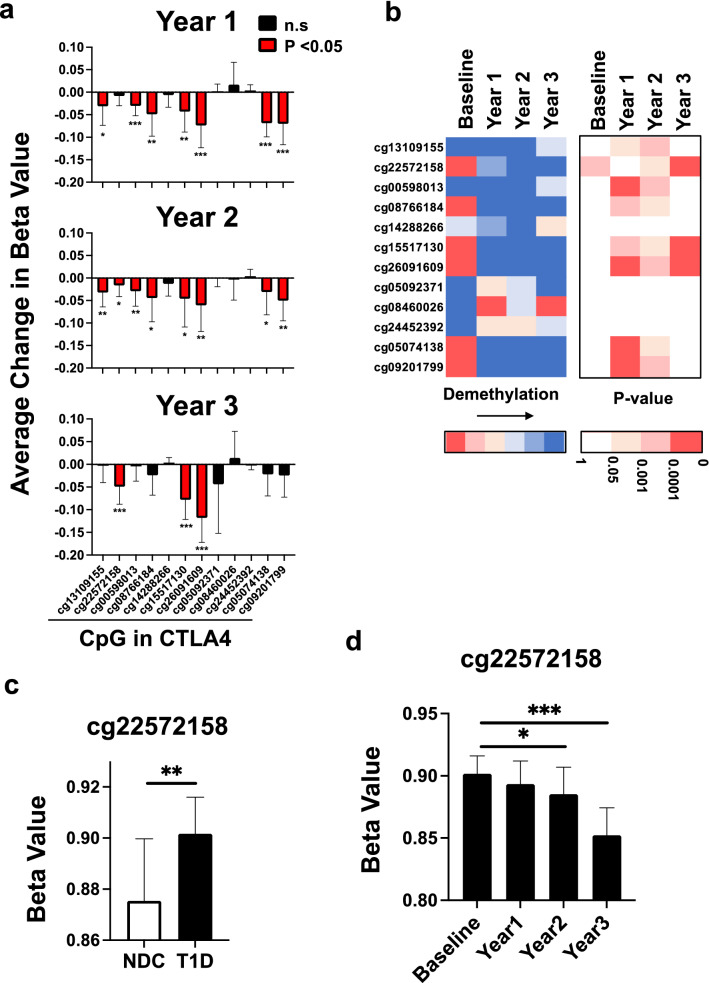
The Treg associated CTLA4 gene of potent Treg cells after in vivo BCG treatment shows gradual, significant and multi-year decrease in methylation after multi-dose BCG therapy. (**a**) Methylation changes for the individual CpG sites of the CTLA4 gene after BCG vaccine therapy were quantified compared to baseline. Average yearly changes in Beta values of CTLA4 CpG sites is shown at Year 1, Year 2 and Year 3 for multi-dosed type 1 diabetic subjects (n = 13). Beta value differences were calculated for each patient relative to baseline. (**b**) The heat map shows average difference in Beta values for individual CTLA4 CpGs sites at yearly intervals. Increased blue coloration in the heatmap means increased demethylation of the CpG sites within the CTLA4 gene. The data for each CpG is color coded separately. The right heatmap indicates the p-values in the corresponding CpG sites; Blank and red indicate without or with significance, respectively, and degree of red color reflects the strength of significance. (**c**) Comparison of Beta values for CD4 + T cells of T1D at baseline versus NDC for the CTLA4 CpG site cg22572158 (known to be associated with diabetes risk) shows that the T1D is over-methylated as compared to control (p = 0.005; unpaired, 2-tailed; T1D n = 13 and NDC n = 8). (**d**) Comparison of Beta values for cg22572158 in T1D CD4 + T cells (n = 13) after treatment with BCG over 3 years time. Asterisks indicate level of significance: p value < 0.05: *; p value < 0.01: **; p value < 0.001: ***. Red bars represent statistically significant change in methylation.

We also looked in detail at the methylation states of CTLA4 CpGs (Fig. 5). In contrast to Foxp3 and CD25, for which demethylation happened gradually, peaking at year 3, the demethylation of CTLA4 CpGs is mostly complete by year 1. CTLA4-cg22572158 is of particular interest because it is known to be associated with diabetes risk^31^ and it is worth noting that this particular CpG was over-methylated at baseline as compared to Non-Diabetic Controls (Fig. 5c) and then gradually demethylated during BCG therapy, with the largest demethylation in year 3 (Fig. 5d). This might suggest this at risk marker of type 1 diabetes is controlled by over-methylation and corrected here in vivo by BCG therapy.

### BCG treatment activates Tregs in vivo, following CD45 Treg activation, CD45RA to CD45RO conversion

**Figure 6:**
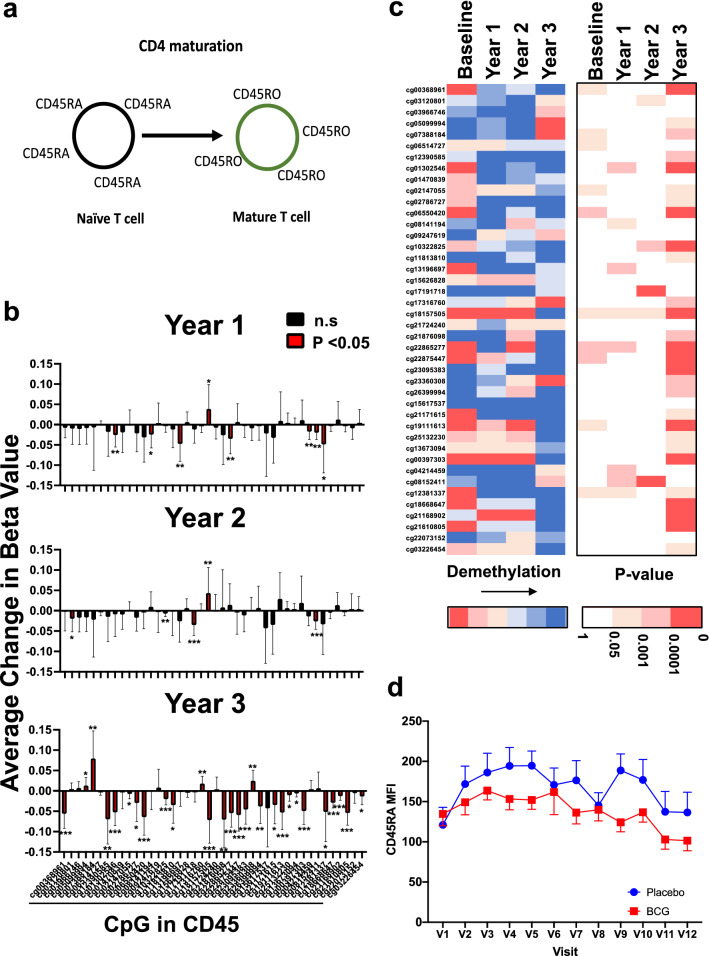
The Treg-associated CD45 gene of activated Treg cells gradually shows decreased methylation after multi-dose BCG vaccine therapy. (**a**) The CD45 protein in naive T cells is the CD45RA splice variant and the CD45RO protein is the splice variant of mature Tregs. (**b**) Methylation changes for the individual CpG sites (n = 34) of the CD45 gene after BCG vaccine therapy are shown as difference in beta values over the three year time course after multi-dose BCG therapy. Data at baseline shows the beginning state of methylation and then the methylation patterns at year 1, year 2 and year 3. The data for each CpG is color coded separately (p value < 0.05: *; p value < 0.01: **; p value < 0.001: ***). Red bars represent statistically significant change in methylation. (**c**) The left heatmap shows average difference in Beta values for individual CD25 CpGs sites at yearly intervals. Increased blue coloration in the heatmap means increased demethylation of the CpG sites within the CD45 gene. The right heatmap indicates the p-values in the corresponding CpG sites; Blank and red indicate without or with significance, respectively, and degree of red color reflects the strength of significance. (**d**) It is common practice to monitor Treg maturation using flow cytometric methods and with antibodies specific to only CD45RA or CD45RO proteins. A longitudinal flow cytometer phenotyping of the cell surface density of naïve CD45RA proteins on CD4 + T cells with APC-CD45RA antibody was performed. The data shows that with BCG therapy (red line) the naïve CD4 + Tregs (CD45RA) cells gradually decreased while the untreated diabetic CD4 T cells placebo had CD45RA naive cells (blue line) that did not decrease over the 3 year monitoring period. At the end of year 3 of monitoring, BCG treated patients have consistently lower CD45RA mean fluoresce intensity (MFI) as compared to placebo which means that the naive cells are decreasing with CD45 cellular maturation. Student’s T-testing over the whole time period shows that the overall difference is statistically significant (unpaired, 2-tailed p = 0.003).

We next asked whether the Tregs in vivo were activated from a naïve state after BCG treatment. Prior published data have shown both by phenotyping for both Foxp3 and for CD25 surface markers and by functional in vitro studies, Tregs are more activated after in vivo BCG therapy and data suggests this frequently uses the TNFR2 receptor (TNFRSF2)^29^. It is known that in CD4 + T cells the CD45 surface marker flips from CD45RA in naïve CD4 + T cells to CD45RO in mature CD4 + T cells (Fig. 6a). In Type 1 diabetes a baseline defect in the CD4 + T cells, including the poorly potent Tregs is too many immature CD45RA cells^27,28^. The CD45 gene is significantly demethylated very early after BCG therapy (year 1) and continues to be demethylation through year 3 (Fig. 1). The demethylation of the CD45 gene is broadly apparent at many CpG sites (Fig. 6b,c).

Another method to confirm that the demethylation of CD45 gene was associated with increased protein expression is to perform flow cytometric studies. In this case, CD45RA naïve Treg cells convert to CD45RO mature cells; we therefore performed flow cytometry to observe the possible disappearance of the naïve T cell marker with the time course of BCG therapy over a 3 year observation period (Fig. 6d). A reduction in CD45RA is equivalent to an increase in CD45RO. Similar to the demethylation data, the flow data showed the continuous loss of CD45RA marker, measured at the mean florescent intensity (MFI), over the 3 years of monitoring of BCG-treated compared to untreated placebo T1Ds (Fig. 6d). Student’s T-test over the whole time period shows that the overall difference is statistically significant (unpaired, 2-tailed p = 0.003). For this marker and with this analysis at the protein level, as early as 8 weeks after the first BCG vaccination, protein changes for this marker could be observed (Visit 2) (p = 0.05).

### Other Treg genes to consider

Other Treg genes in a statistically significant manner de-methylated with in vivo BCG vaccine therapy in type 1 diabetic subjects. This data is shown in heat map format for TNFR2, IKZF4, IKZF2; these genes were part of the original Treg signature genes studied in this paper (Fig. 1). Some Treg studies also investigate additional genes such as ICOS, CCR7, CD28, CD127 and SLAMF1 as important for Treg biology; these genes also de-methylated over the 3 year time course (Supplementary Figs. S3–S5). Three Treg signature genes studied in detail in this paper did not de-methylate (TNFRSF18, CD62L, Fas) over the 3 year observation period and their heat maps of individual CpG regions are shown (Supplementary Fig. S6). Also some Treg biology focus on CCR5, CCR6 and CXCR3 and these genes also overall did not show de-methylation patterns after in vivo BCG vaccinations (Supplementary Figs. S3, S6).

### BCG vaccination impacts mRNA transcripts of Treg genes, a pattern consistent with the de-methylation trends of the same Treg genes

**Figure 7:**
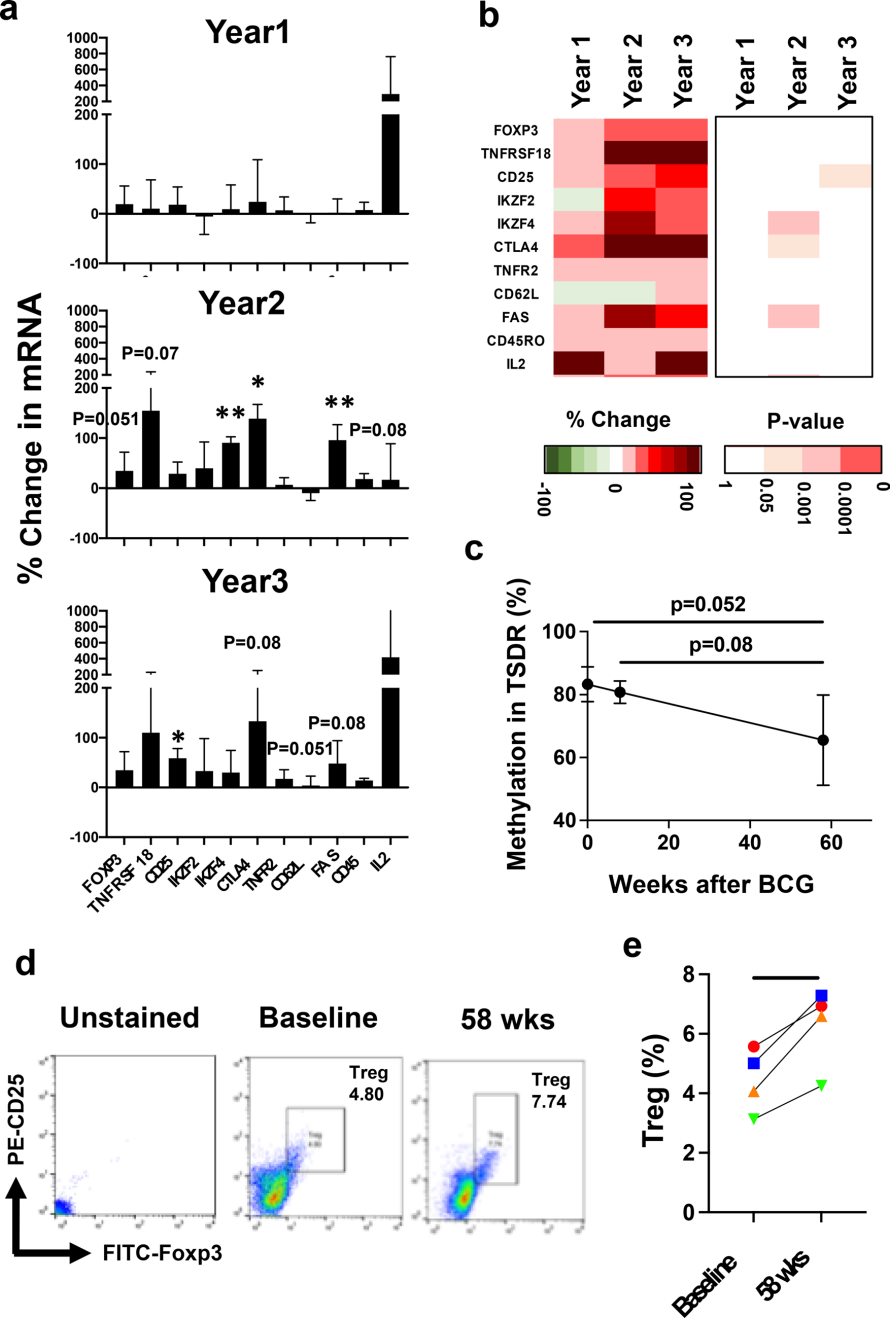
The change in mRNA expression and Treg population by BCG vaccination; direct data on the TSDR region of the Foxp3 gene and quantification of Foxp3 Treg cells. (**a**) The change in transcripts in CD4 + T cell by BCG was evaluated with mRNA seq. While no prominent changes were shown at year 1, most of Treg genes including FOXP3, TNFRSF18, CD25, IKZF4, CTLA4, FAS and CD45 were significantly upregulated at year 2 and year 3. Meanwhile, IKZF2, CD62L and IL2 showed upregulated mRNA expressions without significant. (**b**) The left heatmap indicate overall change in mRNA expression after BCG vaccination in each Treg gene. Red and green indicate up- and down-regulation in mRNA, respectively. (**c**) The average change in methylation (%) in Treg specific demethylated region (TSDR) was shown at baseline, 8 and 58 weeks after BCG vaccination (T1D, n = 4). While the ratio was 83.3 ± 5.5% at baseline, 80.8 ± 3.4% at 8 weeks and 65.5 ± 14.4% at 58 weeks (at 58 weeks, vs. baseline, p = 0.052; vs. 8 weeks, p = 0.08; one-tailed, paired t-test). (**d**) The representative gating images in flow cytometry are shown. Images of unstained, from one patient at baseline and 58 weeks after BCG vaccination are displayed in right, middle and right, respectively. (**e**) The percentage of Treg population was analyzed by flowcytometry. The individual data points for Treg frequency pre- and post-therapy were shown (n = 4). While the average ratio was 4.4 ± 1.1% at baseline, it was 6.3 ± 1.4% at 58 weeks (p = 0.01, two-tailed, paired t-test).

Next, we accessed if BCG vaccinations also affected the mRNA transcripts in the Treg genes that were being demethylated by the introduction of the BCG microbe. While most genes except for IL-2 showed subtle changes at year 1, their transcriptions were clearly upregulated at year 2 (Fig. 7a,b and Supplementary Table). FOXP3, CD25, IZF4, TNFR2 and CD45, all demethylated at year 2 and/or 3, and all exhibited increased mRNA expressions at similar serial time points. The TNFSFR18, CD62L and FAS genes did not show significant demethylation by BCG and did not show mRNA changes. Other Treg genes such as ICOS, CCR6, CD28, and SLAMF1 all promoted their transcript levels at year 2 and/or 3 (Fig. 7a,b and Supplementary Fig. S7). Thus, the majority of Treg genes showed increased mRNA expression patterns after BCG, corresponding to the demethylation by the microbe.

We also investigated the change of methylation in the TSDR and subsequent change in Treg population by BCG vaccinations. Stable Foxp3 expression is associated with epigenetic modification of this TSDR associated with more sable Foxp3 gene expression and more long lasting Treg stability. This DNA region was not part of the Human Infinium Methylation EPIC Bead Chip of DNA sites and therefore required separate study. The TSDR site at baseline in T1D patients showed 83.3 ± 5.5%. After BCG vaccinations, the average methylations were reduced to 80.8 ± 3.6% at 8 weeks and 65.5 ± 14.4% at 58 weeks (Fig. 7c). This increasing demethylation pattern is clear when compared between the percentage at baseline and those at 58 weeks (p = 0.052). Furthermore, we evaluated the Treg population in these vaccinated patients by flow cytometry. Figure 7d shows the representative gating strategies. We found that average Treg population were significantly increasing in number at 58 weeks compared to those at baseline (4.4 ± 1.1 vs. 6.3 ± 1.4%, p = 0.01, Fig. 7e). Taken together, these results suggested that BCG vaccination can demethylate many Treg-related CpG sites including the controlling TSDR for long-term and long term Treg induction in T1D patients.

## Discussion

Treg cells are of central important to immune balance. Restoration or promotion of Treg activity could have meaningful clinical applications for combating autoimmune diseases or inflammatory diseases. One of the challenges of Treg induction or treatment is the chronicity of some diseases such as autoimmune diseases, so therapies need durability and safety. In this study we analyze the possible long-term induction of Tregs in humans with autoimmune diabetes treated with multi-dose BCG therapy. We mechanistically tested the assumption that BCG, like tuberculosis itself and many other chronic infections, promotes Treg expansion on a chronic basis. Furthermore we tested the mechanism of action of BCG on Treg induction at the level of demethylation of key Treg signature genes. We show a gradual and a 3-year time course of BCG vaccine therapy demethylating the signature Treg genes known to be associated with Treg potency. The specific genes studied in this study were Foxp3, TNFSF18, CD25, IKZF2, IKZF4, CTLA4, TNFR2, CD62L, FAS, CD45 and IL2. Foxp3, one of the most essential Treg genes was central to the BCG effect. The Foxp3 gene was over methylated at baseline in type 1 diabetes and over 3 years became demethylated to a level close to non-diabetic controls. The epigenetic effects of the BCG vaccination reported here at the level of DNA of key genes were supported as functionally important since the directional changes were reflected with increased overall mRNA and increased numbers of Treg cells. Clinically and as previously reported, type 1 diabetic subjects respond to repeat BCG vaccinations by a clinically meaningful drop in HbA1c values^12^ (Supplementary Fig. S8). This multi-level data in total supports a role of Treg induction as a possible treatment for the previously observed beneficial effects of this vaccine in clinical trials in autoimmunity.

Although BCG was originally developed as a vaccine for tuberculosis prevention, current clinical trials are using BCG therapy across a broad array of human disease. Not only are there advanced clinical trials with BCG in autoimmune diabetes, but there are also Phase III clinical trials in multiple sclerosis and early stage trial in allergies^6–15^. Epidemiology data also suggests that the unwanted inflammation of Alzheimer’s disease may be responsive to BCG vaccination in adulthood; clinical trials have just begun^32^. Trials using BCG vaccines to lessen the impact of COVID-19 on mortality and morbidity are also underway (clinicaltrial.gov). These ongoing infectious disease trials are based on nearly 10 years of clinical trial data showing BCG not only boosts immune resistance to tuberculosis but also boosts immunity to unrelated infections such as viral respiratory infections, parasites, yellow fever and malaria^33–38^. It is known that BCG vaccines certainly have an impact of immediate cytokine responses and have a strong influence on innate immunity and biological pathways in monocytes as part of the acute innate immune response. For protection against infectious diseases, the time course has been suggested to be short. The innate immune response triggered by BCG, which includes cytokine regulation and innate immunity in monocytes, would be expected to be rapid and to confer broad protection against infections. It is of interest mechanistically that the effects of BCG on autoimmunity take years to manifest clinically, take years to manifest in the T cell compartment, and are durable and gradual at the DNA epigenetic level. Our new data suggests the time course of an adaptive T cell role contrasts with the acute innate role for infection protection from BCG administration.

The data here substantiates a growing view that the impact of BCG vaccines extends to the adaptive immune response. T cells are part of the adaptive immune response. The effects on Treg biology are barely visible for many genes at early evaluation time points (one year) in contrast to genes involved in innate immune responses. Murine and even human data suggest that years after BCG vaccines the bacterium can impact bone marrow, sites of mesenchymal stem cells^21,39^. The reset of stem cells in the primary lymphoid organs suggests adaptive immunity maybe a more central immune mechanism. The theory of a systemic reset of the immune system certainly correlates with autoimmune disease treated with BCG therapy; consistent clinical data shows it takes years for meaningful clinical effects^10,12^. This is not the time course for a sole innate immune response from peripheral exposures. Many would argue that the global rise in autoimmune disease is from too little microbial exposures with our clean modern societies, the well-known Hygiene Hypothesis^40^.

Aggressive type 1 diabetes with rapid pancreatic islet destruction occurs in children with early age of onset; a more indolent form of diabetes occurs with age of onset in adulthood, still with insulin dependence and autoantibodies^30^. If the synergy with microorganisms could change this time course, this study shows that early onset diabetics has time-wise a much more rapid response to BCG in terms of Treg demethylation than the more indolent form of autoimmune diabetes in older adults. This is the trend observed in this study. Previously published data suggests the age of onset of diabetes may be influenced by the timing and the number of child BCG vaccinations^41^. The role of epigenetics in T1D is recognized as a reason why identical twins can have less than 50% concordance of disease and select genes have been associated with hypermethylation in the affected twin pair^42,43^. The presence of de-methylation with BCG and the time course of Treg demethylation in this study is consistent with these observations and mechanisms. Most autoimmune disease show discordance in twin pairs suggestive of a supportive role of epigenetics in human autoimmune diseases^44^. Also the value of microbe exposures through vaccinations might be controlled in part by epigenetics and these studies extend beyond the BCG microbe effect to other various vaccines and infection controlling the host immune system^45^.

Both innate and adaptive immunity can involve the impact of microorganism on protection through epigenetic imprinting, likely with very different gene sets for the acquired resistance and also the immunologic memory of both processed. The innate impact on memory was first demonstrated in insects and plants; systemic acquired resistance translated to protection with reinfection and was at the level of epigenetics^46,47^. It is now well-established for the acute effects of the BCG vaccinations in mice and humans. Often studies using LPS (lipopolysaccharide) for gram negative bacteria show immunity can occur for days and weeks and involves epigenetics. This also related to innate immunity working through NOD1 medicated signaling^48^. Short term 3 months effects of BCG are also associated with epigenetic reprogramming on innate immunity (monocytes) at the level of histones such as H3K4 methylation patterns^49^. The time course we see here on Treg biology is gradual and sustained, but gradually increasing over the passage of time with a time frame of years.

With the recognition that Treg cells can be potent suppressor cells of the immune response, diverse approaches have been tried in the clinic to achieve enhanced Treg effects in vivo therapeutic efficacy. No specific Treg therapy has been approved by a regulatory authority. Human autoimmune diseases could benefit. Studies have investigated the feasibility of using Treg expansion in vitro from autologous cells followed by re-infusion of the expanded Tregs^50^. These adoptive Treg therapy experiments establish the high desirability of disease states needing more Tregs but also present the challenges of expansion with enough autologous Tregs for a treatment, i.e., durability and potency of cell populations after transfer are also a factor. Direct in vivo approaches include the IL2 ligand that works through the CD25 receptor or TNF that works through the TNFR1 and TNFR2 receptors for Treg expansion^51,52^. The challenge with IL2 or TNF ligand therapy are the short in vivo half-life (T_1/2_) of these small proteins and these ligands require careful dosing to prevent toxicity^49,50^. In mouse studies, TNFRp75 deficiency improves mouse survival after tuberculosis exposures and is associated with increased control of bacterial growth during acute and chronic stages of infection^53^. Lack of the TNFR2 receptor would prevent the organism from inducing the most potent human Tregs. It is known TNFR2 expressing Tregs are highly suppressive, consistent with BCG vaccines in vivo also inducing this protein^54^. At baseline, the Treg activation defect, which is found in type 1 diabetes, can be corrected in vitro with TNFR2 agonism^29^. In this current study, by year 3, the TNFR2 receptor is also demethylated with BCG vaccine introduction. Future approaches to inducing Tregs in vivo could be by agonistic antibodies to the TNFR2 receptor^54,55^.

## Conclusion

We show multi-dose BCG vaccinations result in systemic long-term induction of potent Tregs in humans with an underlying autoimmune disease. The clinical time course of BCG-related improvements (2–3 years post-vaccination) correlates with the slow Treg induction through epigenetic modifications. This was manifest here by a general pattern of demethylation of key signature genes of an immunosuppressive Treg cell. In autoimmunity this represents a restoration of Treg biology to near normal expression levels thus demonstrating microorganism synergy and a relatively simple albeit slow and steady approach to the restoration of Treg function in humans. This data supports a role for BCG host microbe interactions on adaptive immunity using methylation mechanisms and beneficial and sustained microbe modifications of host Treg cells.

## Methods

### Research study participants

All human studies had full institutional approvals through Human Research Committee/Institutional Review Board at Partners Healthcare which reviews all protocols for the Massachusetts General Hospital (Study# 2007P001347, 2012P002243 and 2013P002633)^12,20^. The BCG interventional studies were also formally approved by the FDA (IND#2007P001347 and IND#2013P16434). All blood donors, both T1D and non-diabetic control subjects consented through Study #2001P001379. Informed consent was obtained from all subjects and the experiments conformed to the principles set out in the WMA Declaration of Helsinki and the Department of Health and Human Services Belmont Report. The timing of the serial blood sampling times for visits were screening (pre-BCG vaccine), year 1, year 2 and year 3. This study included 13 open-label T1D subjects with 20 years of disease duration. All BCG vaccine treated subjects participated in open-label studies or were unblinded by their participation in earlier Phase I clinical trials. Patients were given two initial intradermal BCG vaccinations at baseline and a second vaccine four weeks later. Non-diabetic control subjects (n = 8) were also studied and compare against T1D patients. The control subjects had a mean age of 26 years. The T1D have a mean age of 45 years and age of disease onset of 24 years.

### Isolation of CD4 + T cells

Blood from BCG treated T1D patients was collected in purple top tubes (K_2_EDTA anti-coagulant) and CD4 + T cells were isolated using magnetic EasySep Direct Human CD4 + T Cell isolation kits from Stem Cell Technologies (Vancouver, BC, Canada, Cat# 19662), following the instructions of the manufacturer as previously described^12,20,25^. Briefly, 1200 μL of Isolation Cocktail and 1200 μL of Rapid Spheres were mixed with 24 mL of whole blood in a 50 mL centrifuge tube and incubated for 5 min at room temperature. PBS (24 mL) was then added and the tube mixed and placed into an “Easy 50” magnet (Stem Cell Technologies, Cat# 18001). This immobilized the unwanted non-CD4 cells at the side of the tube. After 10 min, the CD4-enriched cell suspension was then transferred into a new tube and the magnetic separation process repeated for 5 min with 1200 uL of fresh Rapid Spheres. The cell suspension was then transferred into a new tube and purified for a third time using the magnet. The resulting final CD4 + T cell preparations had purities of > 90% (Supplementary Fig. S1).

### Phenotyping using flow cytometry

Isolated CD4 + T cells were washed in PBS and resuspended in PBS with 2% FBS at a density of 3 × 10^5^ cells/100 μL. The cells were then stained with fluorescent antibodies against surface markers (CD25, CD45RA), washed and fixed and permeabilized for intracellular staining of FoxP3 (BioLegend FoxP3 Fix/Perm Buffer Set, Cat# 421403)^25^. The cells were then analyzed on a FacsCalibur Flow Cytometer (BD Biosciences, San Jose, CA). Antibodies used were PE anti-human CD25 (Biolegend, Clone BC96, Cat# 302606), Alexa Fluor 488 anti-human FoxP3 (BioLegend, Clone 259D, Cat# 320212) and APC anti-human CD45RA (BD Pharmingen, Clone HI100, Cat# 550855).

### Methylation studies

BCG-treated T1D subjects were studied at several time points before and after BCG administrations for changes in methylation of their peripheral blood CD4 + T cells as described previously^20^. These T1D subjects had blood drawn for CD4 + T cells and DNA prepared and stored at − 80 °C. Methylation analysis was performed by the Partners Personalized Medicine Translational Genomics Core. For each sample, 1 μg of DNA was bisulfite-converted using the EZ-96 DNA Methylation Kit (Shallow Well Format) (Zymo Research Corporation, Irvine, CA) following the manufacturer’s protocol with modifications in incubation conditions as recommended for the Infinium Methylation Assay. To verify the quality of bisulfite conversion assay, standard PCR of the highly methylated P19 gene was performed using primers targeting known methylation sites. These primers will anneal to these sites only if the bisulfite conversion is successful. The primers for the methylated targets are: Bisulfite_QC.F (CTAAAACCCCAACTACCTAAA) and Bisulfite_QC.R (TAGGTTTTTTAGGAAGGAGAG). PCR cycling conditions are 95 °C for 15 min, 5 cycles of 95 °C for 30 s, 60 °C for 2 min, 72 °C for 1 min, 30 cycles (of 95 °C for 30 s, 65 °C for 1 min, 72 °C for 1 min), and 72 °C for 5 min. PCR products were run on a 2% agarose gel and a single band at 286 bp indicated successful conversion.

The methylation assay was then performed on the Human Infinium Methylation EPIC Bead Chip Kit (Illumina, San Diego, CA) using 200–400 ng of bisulfite- converted DNA product for each sample according to the Infinium HD Methylation Assay protocol (Illumina, San Diego, CA). This assay targets over 850,000 known and potential methylation sites across the genome via 50-mer probes attached to the Infinium Bead Chips. The bisulfite converted DNA was hybridized to the probes on the Bead Chip followed by a single base extension to incorporate a fluorescent-labeled nucleotide (Cy3 or Cy5) at the target position to differentiate methylation status. The Bead Chips were then imaged on the Illumina iScan reader and the methylation level of each CG locus was calculated in the Genome Studio Methylation module as methylation beta value (beta value = intensity of the methylated allele (M)/(intensity of the unmethylated allele (U) + intensity of the methylated allele (M) + 100). The Illumina CG Database Identifiers (CGs) and corresponding beta values for various genes were then filtered out using fgrep and analyzed using Excel and R.

Prior to settling on the Human Infinium Methylation EPIC Bead Chip, pilot studies were performed comparing various DNA based methods to quantify Treg cells through methylation methods. Those methods included MS-PCR (methylation specific PCR), MS-HRM (methylation specific high resolution melting), pyrosequencing (pyro) and targeted next generation sequencing. All DNA methods of quantifying methylation were compared to the traditional methods of studying Tregs using flow cytometry. All the above methods in our studies were inferior (less quantitative) to flow or to the Human Infinium Methylation EPIC Chip for most DNA sites, so the majority of the data presented in this study only used the Illumina technology. Since the Human Infinium Methylation Epic Bead Chip did not contain the DNA for the TSDR region of the Foxp3 gene, both pyrosequencing and NGBS methods were used to assess methylation of this region (EpigenDx, Hopkinton, MA). With these site specific methylation studies of the TSDR region we used human Foxp3 intron 1 TXDR region (CNS2), through transcript ID; ENST000000376207 with these primers i.e. − 2330 to − 2263 from ATG and + 3997 to + 4064 from TSS, ChrX:49260835–49260768.

Although many genes contribute to the potent Treg phenotype with these cells having maximal suppression, in the human we chose these 11 signature genes to study in this paper. These genes were chosen from the published literature as representing “universal” signature genes of human Tregs^56,57^.

To obtain a core set of Treg signature genes, genes from four sets of data were combined to represent characteristic Treg genes. Those four information sets were: Marodon, G. The meta-Treg signature generated in silico is centered on IL2, members of the TNF superfamily and the endogenous opioid pathway bioRxiv (2019) 10.1101/638072, Pesenacker et al. A regulatory T cell gene signature is a specific and sensitive biomarker to identify children with new onset type 1 diabetes, Diabetes (2019), IPA (Ingenuity Pathway Analysis—Qiagen Digital Analysis database of T cell receptor signaling pathway for Tregs, Okubo, Y et al. Homogeneous expansion of human Treg cells via TNFR2, Scientific Reports (2013). The overlapping data set was Foxp3, TNFRSF18, CD25, IKZF2, IKZF4, CTLA4, TNFR2, CD62L, FAS, CD45 and IL2. Although not part of this complete overlapping signature gene set, some additional Treg associated genes were also present as supplemental information: ICOS, CCR7, CD28, CD127, CXCR3, CCR6, CCR5, CD62L since these genes were listed in > 2 references.

### Data analysis in methylation

Beta values were used to directly quantify methylation levels at each CG site as previously described^20^. Changes in methylation were calculated for each BCG treated patient using beta values on an individual CG level relative to baseline. These individual patient differences in methylation were then averaged at yearly time point after initial BCG doses. These yearly average changes at individual CpG sites were then averaged for each Treg associated gene to quantify the overall yearly methylation changes after BCG. This analysis was also repeated after separating the patients according to diabetes age of onset (AOO) in which patients with an AOO below 20 years old (n = 5) and patients with an AOO above 20 years old (n = 8) were placed in separated groups. Foxp3 was studied in two ways. Type 1 diabetics compared to non-diabetic controls at baseline prior to any treatment and then like other critical Treg genes as a comparison to self over the 3 year observation period. The individual CpG analysis was also conducted by separated the diabetic patients according to AOO as mentioned.

### Data analysis in RNA sequencing

As previously described, Total RNA was extracted from fleshly isolated CD4 + T cells using the RNeasy Plus Mini Kit (QIAGEN), and RNA sequencing was performed at the BioMicro Center (BMC) of the Massachusetts Institute of Technology and the Center for Cancer Computational Biology (CCCB) of the Dana-Farber Cancer Institute (DFCI)^12,20^. The quality of the extracted mRNA was determined with an Agilent 2100 bioanalyzer. The poly A selection was performed with a NEBNext Poly(A) mRNA Magnetic Isolation Module and the extracted mRNA was used for library preparation with the NEBNext UltraTM Directional RNA Library Prep Kit for Illumina. The RNAseq libraries were run on a high sensitivity DNA chip on the Agilent 2100 Bioanalyzer and the final concentration of 2 pM of libraries were loaded onto the Illumina NextSeq 500. TopHat was used for alignment of reads against reference genome HG19. Cufflinks was used for the analyses and the data then was normalized with DESeq (part of the R, Bioconductor package). The resulting raw data were also normalized by average value in the mRNA expressions of multiple housekeeping genes including *ATCB*, *GAPDH*, *PGK1*, *YWHAZ*, *SDHA*, *TFRC*, *GUSB*, *HMBS* and *TUBB* for the calibration among sequences prior to the analyses. Then the normalized value was compared and exhibited as the percent change between NDC and T1D at baseline or as T1D at baseline and 1–3 year after BCG vaccinations.

## Supplementary Information

Supplementary Information 1.

Supplementary Information 2.

## Abbreviations

Tregs: T-regulatory cells
GVHD: Graft versus host disease
BCG: Bacille–Calmette–Guérin
MS: Multiple sclerosis
TB: Tuberculosis
T1D: Type 1 diabetes
NDC: Non-diabetic control
AOO: Age of onset
MFI: Mean fluorescent intensity
TSDR: T-regulatory cell specific de-methylated region

## References

[1] Belkaid Y, Rouse BT (2005). Natural regulatory T cells in infectious disease. Nat. Immunol..

[2] Boer MC, Joosten SA, Ottenhoff TH (2015). Regulatory T-cells at the interface between human host and pathogens in infectious diseases and vaccination. Front. Immunol..

[3] Parente JNT (2015). T regulatory cells distribution in the different clinical forms of leprosy and reactional states. Ann. Bras. Dermatol..

[4] Cardona P, Cardona PH (2019). Regulatory T cells in *Mycobacterium tuberculosis* infection. Front. Immunol..

[5] Jaron B, Maranghi E, Leclerc C, Majlessi L (2008). Effect of attenuation of Treg during BCG immunization on anti-mycobacterial Th1 responses and protection against *Mycobacterium tuberculosis*. PLoS ONE.

[6] Bennett CL (2001). The immune dysregulation, polyendocrinopathy, enteropathy, X-linked syndrome (IPEX) is caused by mutations of FOXP3. Nat. Genet..

[7] Hori S, Nomura T, Sakaguchi S (2003). Control of regulatory T cell development by the transcription factor Foxp3. Science.

[8] Ristori G (1999). Use of Bacille Calmette-Guerin (BCG) in multiple sclerosis. Neurology.

[9] Paolillo A (2003). The effect of Bacille Calmette-Guerin on the evolution of new enhancing lesions to hypointense T1 lesions in relapsing remitting MS. J. Neurol..

[10] Ristori G (2014). Effects of Bacille Calmette-Guerin after the first demyelinating event in the CNS. Neurology.

[11] Faustman DL (2012). Proof-of-concept, randomized, controlled clinical trial of Bacillus-Calmette-Guerin for treatment of long-term type 1 diabetes. PLoS ONE.

[12] Kuhtreiber WM (2018). Long-term reduction in hyperglycemia in advanced type 1 diabetes: The value of induced aerobic glycolysis with BCG vaccinations. NPJ Vaccines.

[13] Kuhtreiber WM, Faustman DL (2019). BCG therapy for type 1 diabetes: Restoration of balanced immunity and metabolism. Trends Endocrinol. Metab..

[14] El-Zein M, Parent ME, Benedetti A, Rousseau MC (2010). Does BCG vaccination protect against the development of childhood asthma? A systematic review and meta-analysis of epidemiological studies. Int. J. Epidemiol..

[15] Choi IS, Koh YI (2002). Therapeutic effects of BCG vaccination in adult asthmatic patients: A randomized, controlled trial. Ann. Allergy Asthma Immunol..

[16] Koeken VA (2020). BCG vaccination in humans inhibits systemic inflammation in a sex-dependent manner. J. Clin. Invest..

[17] Arts RJW (2016). Immunometabolic pathways in BCG-induced trained immunity. Cell Rep..

[18] Arts RJ (2016). Glutaminolysis and fumarate accumulation integrate immunometabolic and epigenetic programs in trained immunity. Cell Metab..

[19] Kleinnijenhuis J (2012). Bacille Calmette-Guerin induces NOD2-dependent nonspecific protection from reinfection via epigenetic reprogramming of monocytes. Proc. Natl. Acad. Sci. USA.

[20] Kuhtreiber WM (2020). BCG vaccinations upregulate myc, a central switch for improved glucose metabolism in diabetes. Science.

[21] Kaufmann E (2018). BCG educates hematopoietic stem cells to generate protective innate immunity against tuberculosis. Cell.

[22] Schmidl C, Delacher M, Huehn J, Feuerer M (2018). Epigenetic mechanisms regulating T-cell responses. J. Allergy Clin. Immunol..

[23] Floess S (2007). Epigenetic control of the foxp3 locus in regulatory T cells. PLoS Biol..

[24] Polansky JK (2008). DNA methylation controls Foxp3 gene expression. Eur. J. Immunol..

[25] Okubo Y, Mera T, Wang L, Faustman DL (2013). Homogeneous expansion of human T-regulatory cells via tumor necrosis factor receptor 2. Sci. Rep..

[26] Pesenacker AM (2016). A regulatory T-cell gene signature is a specific and sensitive biomarker to identify children with new-onset type 1 diabetes. Diabetes.

[27] Faustman D, Eisenbarth G, Breitmeyer J, Schlossman S (1990). Analysis of T lymphocyte subsets in all stages of diabetes. J. Autoimmun..

[28] Faustman D, Schoenfeld D, Ziegler R (1991). T-lymphocyte changes linked to autoantibodies: Association of insulin autoantibodies with CD4+CD45R+ lymphocyte subpopulation in prediabetic subjects. Diabetes.

[29] Okubo Y, Torrey H, Butterworth J, Zheng H, Faustman DL (2016). Treg activation defect in type 1 diabetes: Correction with TNFR2 agonism. Clin. Transl. Immunol..

[30] Wang L, Lovejoy NF, Faustman DL (2012). Persistence of prolonged C-peptide production in type 1 diabetes as measured with an ultrasensitive C-peptide assay. Diabetes Care.

[31] Ye J (2018). Identification of loci where DNA methylation potentially mediates genetic risk of type 1 diabetes. J. Autoimmun..

[32] Gofrit ON (2019). Bacillus Calmette-Guerin (BCG) therapy lowers the incidence of Alzheimer's disease in bladder cancer patients. PLoS ONE.

[33] Aaby P (2011). Randomized trial of BCG vaccination at birth to low-birth-weight children: Beneficial nonspecific effects in the neonatal period?. J. Infect. Dis..

[34] Elguero E, Simondon KB, Vaugelade J, Marra A, Simondon F (2005). Non-specific effects of vaccination on child survival? A prospective study in Senegal. Trop. Med. Int. Health.

[35] Kristensen I, Aaby P, Jensen H (2000). Routine vaccinations and child survival: Follow up study in Guinea-Bissau, West Africa. BMJ.

[36] Nankabirwa V (2015). Child survival and BCG vaccination: A community based prospective cohort study in Uganda. BMC Public Health.

[37] Hollm-Delgado MG, Stuart EA, Black RE (2014). Acute lower respiratory infection among Bacille Calmette-Guerin (BCG)-vaccinated children. Pediatrics.

[38] de Castro MJ, Pardo-Seco J, Martinon-Torres F (2015). Nonspecific (heterologous) protection of neonatal BCG vaccination against hospitalization due to respiratory infection and sepsis. Clin. Infect. Dis..

[39] Cirovic B (2020). BCG vaccination in humans elicits trained immunity via the hematopoietic progenitor compartment. Cell Host Microbe.

[40] Strachan DP (1989). Hay fever, hygiene, and household size. BMJ.

[41] Karaci, M. in *The Value of BCG and TNF in autoimmunity* (ed D. Faustman) 52–62 (Elsevier, 2014).

[42] Jerram ST, Dang MN, Leslie RD (2017). The role of epigenetics in type 1 diabetes. Curr. Diab. Rep..

[43] Dang MN, Buzzetti R, Pozzilli P (2013). Epigenetics in auotimmune idseases with focus on type 1 diabetes. Diabetes Metab. Res. Rev..

[44] Mazzone R, Zwergel C, Artico M (2019). The emerging role of eptigenetics in human autoimmune disorders. Clin. Epigenet..

[45] Bannister S, Messina NK, Novakovic B (2020). The emerging role of eptigenetics in the immune response to vaccination and infection: A systemic review. Epigenetics.

[46] Rodrigues J, Brayner FA, Alves LC, Dixit R, Barillas-Mury C (2010). Hemocyte differentiation mediates innate immune memory in *Anopheles gambiae* mosquitoes. Science.

[47] Conrath U (2011). Molecular aspects of defence priming. Trends Plant Sci..

[48] Clarke TB (2010). Recognition of peptidoglycan from the microbiota by Nod1 enhances systemic innate immunity. Nat. Med..

[49] Mourits VP, Wijkmans JC, Joosten LA, Netea MG (2018). Trained immunity as a novel therapeutic strategy. Curr. Opin. Pharmacol..

[50] Romano M, Fanelli G, Albany CJ, Giganti G, Lombardi G (2019). Past, present, and future of regulatory T cell therapy in transplantation and autoimmunity. Front. Immunol..

[51] Rosalia RA, Arenas-Ramirez N, Bouchaud G, Raeber ME, Boyman O (2014). Use of enhanced interleukin-2 formulations for improved immunotherapy against cancer. Curr. Opin. Chem. Biol..

[52] Beutler B, Milsark IW, Cerami AC (1985). Passive immunization against cachectin/tumor necrosis factor protects mice from lethal effect of endotoxin. Science.

[53] Keeton R (2014). Soluble TNFRp75 regulates host protective immunity against *Mycobacterium tuberculosis*. J. Clin. Invest..

[54] Zou H, Li R, Hu H, Hu Y, Chen X (2018). Modulation of regulatory T cell activity by TNF receptor type II-targeting pharmacological agents. Front. Immunol..

[55] Torrey H (2020). A novel TNFR2 agonist antibody expands highly potent regulatory T cells. Sci. Signal.

[56] Marodon G (2020). In Silico characterization of a “universal” Treg signature reveals the pro enkephalin gene as a novel Treg marker. BioRxiv.

[57] Pfoertner S (2006). Signatures of human regulatory T cells: An encounter with old friends and new players. Genome Biol..

