# Supplementary material for: BCG therapy is associated with long-term, durable induction of Treg signature genes by epigenetic modulation
Source: Sci Rep. 2021 Jul 22;11:14933. doi: 10.1038/s41598-021-94529-2 (PMC8298580; doi:10.1038/s41598-021-94529-2)
Supplement: Supplementary file 1 — Supplementary Information 1. [file 41598_2021_94529_MOESM1_ESM.docx]

**Supplementary information**

**Supplementary Fig. S1. | The purity of fleshly isolated CD4+ T cells.** The CD4+ T cells were isolated from whole blood. Following the protocol, more than 90% of purity was ensured. Left and right images indicate representative cell gating strategies with unstained and stained with APC-conjugated anti-CD4 antibody, respectively.

**Supplementary Fig. S2. | The overall methylation pattern of CpG sites in T1D CD4+ T cell.** In total 697315 CpG sites in CD4+ T cell were evaluated and their methylation pattern were compared. All individual data were displayed in volcano plots; X- and Y-axis indicate difference in beta value and -Log (p-value), respectively. Dots in left and right side of blue line (difference=0) indicate demethylation and over-methylation, respectively. Red line indicates Y=1.301, which means p=0.05. The number of CpG (%) was summarized in bar graphs; Black and white bars indicate hypo- and hypermethylated CpG sites, respectively. At baseline, 49.5% (8.8% with significance) and 50.5% (9.4%) of CpG were hyper- and hypomethylated in T1D, respectively, compared to NDC. After BCG vaccination, 44.1% (3.6%) and 55.5% (3.3%) at year 1, 43.9% (5.6%) and 55.8% (8.5%) at year 2, and 37.3% (24.3%) and 62.6% (48.4%) at year 3 were hyper- and hypomethylated in T1D, respectively, compared to their beta values at baseline.

**Supplementary Fig. S3. | The methylation changes in additional Treg associated genes.** Methylation changes in ten additional Treg signature genes were quantified yearly after multi-dose BCG vaccine therapy (T1D, n=13). The methylation values are current methylation values at year 1, year 2 or year 3 compared to baseline expressed as average change in beta values. Positive beta values indicate increased methylation and thus decreased gene expression; negative methylation values indicate demethylation and increased gene expression. Significant decreases in methylation after BCG were seen in eight of 5 genes: ICOS, CCR7, CD28, CD127 and SLAMF1 (p value < 0.05: * ; p value < 0.01: ******; p value < 0.001: *******). Red bars represent statistically significant change in methylation.

**Supplementary Fig. S4. | The methylation changes in additional Treg associated genes with demethylation represented as individual CpG sites in heatmap format-1.** The Treg-associated genes including TNFR2, IKZF2, IKZF4, SLAMF1 and IL2 all showed by year 3 after in vivo BCG vaccinations, the gradual and statistically significant de-methylation of CpG sites. The left heatmap shows average difference in Beta values for individual CpGs sites at yearly intervals. Increased blue coloration in the heatmap means increased demethylation of the CpG sites. The data for each CpG is color coded separately. The right heatmap indicates the p-values in the corresponding CpG sites; Blank and red indicate without or with significance, respectively, and degree of red color reflects the strength of significance.

**Supplementary Fig. S5. | The methylation changes in additional Treg associated genes with demethylation represented as individual CpG sites in heatmap format-2.** The Treg-associated genes including ICOS, CCR7, CD28 and CD127 all showed by year 3 after in vivo BCG vaccinations, the gradual and statistically significant de-methylation of CpG sites. The left heatmap shows average difference in Beta values for individual CpGs sites at yearly intervals. Increased blue coloration in the heatmap means increased demethylation of the CpG sites. The data for each CpG is color coded separately. The right heatmap indicates the p-values in the corresponding CpG sites; Blank and red indicate without or with significance, respectively, and degree of red color reflects the strength of significance.

**Supplementary Fig. S6. | The methylation changes in additional Treg associated genes without demethylation represented as individual CpG sites in heatmap format.** Some Treg associated genes including TNFRSF18, CD62L, FAS, CCR5, CCR6 and CXCR3 did not show statistically significant de-methylation of their CpG sites over the 3 year observation period. The left heatmap shows average difference in Beta values for individual CpGs sites at yearly intervals. Increased blue coloration in the heatmap means increased demethylation of the CpG sites. The data for each CpG is color coded separately. The right heatmap indicates the p-values in the corresponding CpG sites; Blank and red indicate without or with significance, respectively, and degree of red color reflects the strength of significance.

**Supplementary Fig. S7. | The change in mRNA expressions of additional Treg associated genes after BCG vaccination. a.** While no prominent changes were shown at year 1, four of 8 additional Treg genes including ICOS, CCR6, CD28 and SLAMF1 were significantly or marginally upregulated at year 2 and/or 3. Meanwhile, CCR5, CCR7 and CD127 showed upregulated mRNA expressions without significant, and CXCR3 showed almost no change in mRNA expressions. **b.** The left heatmap indicate overall change in mRNA expression after BCG vaccination in each Treg gene. Red and green indicate up- and down-regulation in mRNA, respectively. The right heatmap indicates the p-values in the corresponding CpG sites; Blank and red indicate without or with significance, respectively, and degree of red color reflects the strength of significance.

**Supplementary Fig. S8. | Long term improvement of blood sugars in T1D after BCG vaccinations; evaluated with HbA1c monitoring.** Previously published data shows the gradual 3 year long time course of BCG vaccination induced drops in HbA1c values (red) compared to two different reference/placebo groups of adult T1D (black)^12^. The data also shows the long term drop in HbA1c values is durable to at least 8 years from trial enrollment. **a.** Data expressed as the % change in HbA1c values from baseline through year 8. **b.** Data is shown with real HbA1c values expressed as %.

**Supplementary Table 1. | Individual processed data in methylation and mRNA sequence. 1.** Average difference in beta value of Treg-associated genes at individual CpG levels are shown. **2.** Average difference in beta value of Treg-associated genes are shown. **3.** Average difference in beta value of Foxp3-associated CpG sites are shown at AOO<20 and 20<. **4.** Average change in mRNA expression of Treg-associated genes after BCG vaccination are shown.
