# Supplementary material for: BCG therapy is associated with long-term, durable induction of Treg signature genes by epigenetic modulation
Source: Sci Rep. 2021 Jul 22;11:14933. doi: 10.1038/s41598-021-94529-2 (PMC8298580; doi:10.1038/s41598-021-94529-2)
Supplement: Supplementary file 2 — Supplementary Information 2. [file 41598_2021_94529_MOESM2_ESM.pdf]

	Year1 (T1D)			Year2
Gene	%Chane in mRNA	SD	p-value	%Chane in mRNA
FOXP3	19.50127015	36.50742	0.242072	34.59816851
TNFRSF18	10.32578447	58.0064	0.489723	154.9810893
CD25	18.53085793	35.58999	0.233205	29.08916377
IKZF2	-5.58378266	36.02088	0.308788	40.01531088
IKZF4	9.327021308	48.63803	0.357391	90.63427962
CTLA4	24.33997233	84.47058	0.324063	139.070804
TNFR2	7.344211275	26.67867	0.291871	6.66308803
CD62L	-0.116886462	18.03592	0.442174	-9.794849105
FAS	2.20121211	27.5281	0.467684	95.97072251
CD45RO	7.794215577	15.42182	0.30067	18.33890951
IL2	294.0863877	467.9602	0.317039	16.99215223
ICOS	11.38578562	42.7907877	0.30429067	31.40010998
CCR5	22.69993603	67.3667016	0.2912086	16.82750696
CCR6	-0.32024458	61.3713794	0.45230875	83.10279513
CCR7	-4.033368331	14.894658	0.37756003	4.758258671
CXCR3	-1.77906242	46.7262825	0.40349406	-1.657759022
CD28	9.57637179	43.3814886	0.3386167	60.41881687
CD127	10.82560644	10.7712586	0.28753568	23.50524783
SLAMF1	13.7091844	37.337345	0.32144789	57.30657963

(T1D)		Year3 (T1D)		
SD	p-value	%Chane in mRNA	SD	p-value
37.29246	0.050944	34.474124	37.33172	0.124551
82.99157	0.072548	110.1413506	119.0558	0.158474
23.08433	0.110999	58.90163936	19.05378	0.037702
52.51302	0.097406	32.85651644	65.36617	0.19115
11.82744	0.003755	29.87897881	44.37441	0.190735
28.10278	0.017282	133.1290995	118.7114	0.079648
14.23047	0.256821	17.32710237	18.28835	0.051613
14.83501	0.209418	3.862866524	18.95792	0.464516
30.81562	0.002865	48.08087014	45.94372	0.080852
10.75559	0.087154	13.78437513	4.524694	0.15853
72.03256	0.399236	417.6596613	614.7531	0.481263
12.8023545	0.0040318	35.44675002	40.2002152	0.05160596
47.0942007	0.25284298	-11.73780008	18.1189707	0.26369178
27.2524676	0.01872684	129.7761284	128.998287	0.08186149
28.424833	0.37933652	26.3992875	30.5861443	0.10960042
40.8078677	0.27959942	-11.07001359	39.1033608	0.15596781
40.1687587	0.01164123	62.12760526	79.4119299	0.1033114
10.2168699	0.16671561	23.59517211	12.5448139	0.15223655
44.983434	0.0511881	40.70303145	47.4531729	0.14940986
